# Identification of genes potentially involved in solute stress response in *Sphingomonas wittichii* RW1 by transposon mutant recovery

**DOI:** 10.3389/fmicb.2014.00585

**Published:** 2014-11-04

**Authors:** Edith Coronado, Clémence Roggo, Jan R. van der Meer

**Affiliations:** Department of Fundamental Microbiology, University of LausanneLausanne, Switzerland

**Keywords:** *Sphingomonas*, water stress, transposon mutants, flow cytometry, bioremediation

## Abstract

The term water stress refers to the effects of low water availability on microbial growth and physiology. Water availability has been proposed as a major constraint for the use of microorganisms in contaminated sites with the purpose of bioremediation. *Sphingomonas wittichii* RW1 is a bacterium capable of degrading the xenobiotic compounds dibenzofuran and dibenzo-*p*-dioxin, and has potential to be used for targeted bioremediation. The aim of the current work was to identify genes implicated in water stress in RW1 by means of transposon mutagenesis and mutant growth experiments. Conditions of low water potential were mimicked by adding NaCl to the growth media. Three different mutant selection or separation method were tested which, however recovered different mutants. Recovered transposon mutants with poorer growth under salt-induced water stress carried insertions in genes involved in proline and glutamate biosynthesis, and further in a gene putatively involved in aromatic compound catabolism. Transposon mutants growing poorer on medium with lowered water potential also included ones that had insertions in genes involved in more general functions such as transcriptional regulation, elongation factor, cell division protein, RNA polymerase β or an aconitase.

## Introduction

Bioremediation rates of organic pollutants can be enhanced by the use of specific microbial strains or consortia that degrade the contaminants of interest (Vogel, 1996; Shi et al., 2001; Ahn et al., 2008; Chen et al., 2008; Das et al., 2008; Rehmann et al., 2008; Kumar et al., 2009). However, successful bioaugmentation is not only dependent on the inherent capacities of the inoculated strain(s), but on a variety of environmental and biological factors as well (Leahy and Colwell, 1990; Holden et al., 1997). One of the main environmental factors controlling activity of introduced strains for bioremediation is thought to be the availability of water (water *activity* or water *potential*) (Holden et al., 1997).

Water stress is a consequence of the lowering of water potential, with less water available to enter the cell and to maintain regular intracellular biochemical processes (Brown, 1976). The water potential has two components, the solute potential (SP) and matric potential (MP). The SP increases linearly with increasing concentration of solutes whereas the MP describes the interaction of water with surfaces and interfaces (colloidal particles and solid particles from 0.002 to 1 μm diameter) (Brown, 1976; Potts, 1994). Cells under solute stress face diminished water potential as a consequence of high concentrations of solutes outside the cell and will experience a net flux of water toward the extracellular environment. Matric stress is a consequence of the net flux of water from the inside to the outside as a result of capillary forces of non-permeating solutes (Potts, 1994). A different approach to manipulate the matric stress that a cell can experience uses a porous surface, as described by Dechesne et al. (2008). This method allows to impose a suction on the porous matrix corresponding to a pre-determined soil MP, while permitting constant microscopic observation of the cells (Dechesne et al., 2008, 2010; Gülez et al., 2010, 2012).

Microorganisms are known to be able to defend themselves against low water potentials by changing their membrane fatty acid composition, synthesizing compatible intracellular solutes such as trehalose or sucrose, producing exopolysaccharides or overproducing transmembrane transporters (Boch et al., 1994; Lucht and Bremer, 1994; Ogahara et al., 1995; Halverson and Firestone, 2000; Hallsworth et al., 2003; Mutnuri et al., 2005; Singh et al., 2005; Reva et al., 2006; Leblanc et al., 2008; Gülez et al., 2010; Brill et al., 2011; Johnson et al., 2011). It is also known that solute and matric stress result in different effects on cells (Halverson and Firestone, 2000; Axtell and Beattie, 2002; Hallsworth et al., 2003; Reva et al., 2006; Cytryn et al., 2007; Johnson et al., 2011; Gülez et al., 2012). In the case of microorganisms with a potential use in bioremediation, their resistance to water stress becomes a high priority if they are to be introduced in the environment (Holden et al., 1997; Leblanc et al., 2008; Johnson et al., 2011). *P. putida* induces alginate biosynthesis genes and genes responsible for trehalose biosynthesis to cope with water stress (Gülez et al., 2012). *Rhodococcus jostii* induces a protective response against oxidative stress and initiates synthesis of the compatible solute ectoine, when exposed to desiccation stress (Leblanc et al., 2008). Johnson et al. (2011) observed 2.5 and 7.2-fold increased expression of two genes for an extracellular sigma24 factor (Swit_3836 and Swit_3924) when exposing *S. wittichii* RW1 to solute stress, and further for genes involved in protein turnover and repair in response to matric stress. Both under solute and matric stress, an induction of *S. wittichii* genes for trehalose, exopolysaccharide and flagella biosynthesis was observed (Johnson et al., 2011).

Sphingomonads are often found in contaminated environments due to their ability to degrade a wide range of xenobiotic compounds, making them an interesting choice for bioremediation (Leys et al., 2004; Peng et al., 2008). Members of this genus can degrade herbicides (Zipper et al., 1996; Kohler, 1999; Keum et al., 2008), pesticides (Manickam et al., 2008) and a wide range of polyaromatic hydrocarbons (PAHs), such as biphenyl (Baboshin et al., 2008), naphthalene (Story et al., 2004), phenanthrene (Tao et al., 2009), chrysene (Willison, 2004), azo dyes (Stolz, 1999) or dioxins (Fortnagel et al., 1990; Wittich et al., 1992; Hong et al., 2002). Among sphingomonads, *S. wittichii* RW1 has been studied extensively for its multiple xenobiotic degrading abilities (Wittich et al., 1992; Happe et al., 1993; Nam et al., 2005; Keum et al., 2008). Several reports have focused on the remarkable capacity of strain RW1 to degrade dibenzo-*p*-dioxins and dibenzofurans (Wittich et al., 1992; Happe et al., 1993; Wilkes et al., 1996; Megharaj et al., 1997; Halden et al., 1999; Hong et al., 2002), making it a suitable candidate to be used for bioaugmentation. The previously reported genome-wide transcription analysis of RW1 exposed or not to solute or matric stress has helped to identify the genes differentially responding to such conditions, but this is not sufficient to unambiguously demonstrate their role in low water resistance (Johnson et al., 2011). For this purpose, gene replacement or gene deletion are better techniques (Martínez-García et al., 2011), however, the construction of targeted gene deletions in RW1 has so far remained elusive. In a different study to detect genes impaired in water stress survival Roggo et al. used sequencing of transposon mutant libraries before and after salt exposure and compared mutant abundances (Roggo et al., 2013). As an alternative, we use here transposon mutagenesis followed by screening for growth differences to actually isolate mutants with disruptions in genes for potential water stress resistance factors. We focus solely on the induction of water stress through increase of external SP. Two mini-Tn5 mutant libraries were created, one using the pRL27 plasposon system (Larsen et al., 2002), and the second one with a modified version, pRL27::miniTn5-*egfp*, coding for a promoterless *egfp* gene. The resulting libraries were screened by three different procedures, which we expected would emphasize specific drought-stress induced differences. The first procedure consisted of replica plating and screening for the absence of growth on NaCl-amended agar plates. In the second procedure we took advantage of the high throughput of flow cytometry (FC) and tested for poorer growth of mutant microcolonies inside agarose beads upon exposure to NaCl-amended medium. Finally, in the third procedure and different mutant library we recovered by fluorescence assisted cell sorting individual mutant cells with higher expression of inserted miniTn5-*egfp* upon NaCl exposure. The insertion sites of the transposon mutants were recovered and determined by DNA sequencing, and mapped onto the RW1 genome. Recovered mutants were regrown in pure culture and their growth rates (and, where relevant, eGFP expression) under normal and NaCl-amended culture medium were compared.

## Materials and methods

### Cultivation of bacteria

A stock of *S. wittichii* RW1 was kept at −80°C and a small aliquot was plated on agar with 5 mM salicylate (SAL). Minimal media was based on DSM457 (German Resource Centre for Biological Material, Braunschweig, Germany) amended with 5 mM salicylate (MM+SAL). Agar plates consisted of MM+SAL supplemented with 1.5% of bacteriological agar No.1 (Oxoid). All RW1 cultures were incubated at 30°C. For selection and maintenance of the transposon insertions, kanamycin (Km, at 50 μg per ml) was added to MM+SAL. *Escherichia coli* strains were grown in Lysogeny Broth (LB) to which Km was added to maintain the selective pressure for the plasposon vectors. *E. coli* was incubated at 37°C according to standard procedures. Tables S1 to S3 show lists of strains, plasmids and primers used here.

### Reduced water potential conditions

Liquid and solid media with lowered water activity (potential) compared to the control media were prepared following the method described by Halverson and Firestone (2000). 17.4 g/L of NaCl were added to achieve a decrease in water potential of −1.5 MPa in comparison to the control (the control media has a water potential of around −0.23 MPa). A stationary phase culture (OD_600_~1.0) of *S. wittichii* RW1 was used to inoculate 50 ml flasks containing 15 ml of MM+SAL (control) and flasks containing NaCl-amended MM+SAL. Three replica flasks were prepared for each condition. Cultures were inoculated at an initial optical density of OD_600_ = 0.005, and incubated on a rotary shaker at 30°C until stationary phase was reached (OD~1). The OD_600_ was measured regularly (Ultrospec, GE) and the maximum specific growth rate (μ_max_, h^−1^) as a function of water potential was calculated by linear regression on ln-transformed OD-values vs. time.

### Transposon mutant libraries

Two different transposon mutant libraries of *S. wittichii* RW1 were created. The first involved the plasposon plasmid pRL27 (Larsen et al., 2002) and the second a modified version, the plasmid pRL27::miniTn5-*egfp*. To produce the first library, 2 ml of *S. wittichii* RW1 and 1 ml of *E. coli* BW20767 (pRL27::miniTn5) overnight cultures were mixed and centrifuged for 2 min at 8000 rpm. The supernatant was discarded and the cell pellet resuspended in 50 μl of sterile saline solution (NaCl 0.9%). The 50 μl droplet was placed on the surface of an LB plate and incubated at 30°C for 16 h. After incubation, the cell layer was recovered with a sterile loop, resuspended in 1 ml saline solution and 150 μl aliquots were plated on selective media (MM+SAL+Km). The plates were incubated at 30°C during several days and when colonies were visible they were picked individually for replicate screening, or washed off to produce a mixed enriched RW1 transposon library.

Plasmid pRL27::miniTn5-*egfp* was constructed by ligating Asp718-digested and 5′end Klenow filled pRL27-DNA to the SmaI-EcoRV *egfp*-containing DNA fragment of pPROBE-GFP-tagless (Miller et al., 2000). The ligation mixture was used to transform *E. coli* BW20767 (Metcalf et al., 1996). A single colony of *E. coli* carrying pRL27::miniTn5-*egfp* was selected, verified for correctness of the plasmid and the orientation of the *egfp* gene, and used for transposon mutagenesis with *S. wittichii* RW1 as described above. Colonies growing on MM+SAL+Km plates were washed off with saline solution and kept as mutant library mix (library 2). The library was divided in 1 ml aliquots, which were stored at −80°C.

### Transposon library screening

The *S. wittichii* RW1 pRL27-generated library was screened for growth impairment by replica plating on medium without or with NaCl (equivalent to a −1.5 MPa decrease in water potential). Individual RW1 colonies were picked from MM+SAL+Km plates and replica-plated in parallel on control plates (MM+SAL+Km) and NaCl-amended MM+SAL+Km agar plates (17.4 g NaCl/L). Colonies that failed to grow on MM+SAL+Km-NaCl but grew on control plates were selected for further characterization.

The mixed pRL27 library of RW1 was also used to screen *en masse* for growth deficiencies in a FC procedure, in which individual cells were encapsulated in agarose beads and incubated in growth medium with lowered water potential by addition of NaCl. Encapsulated cell mixtures were prepared as follows: a single frozen RW1 Tn*5* mutant library aliquot was grown until stationary phase in MM+SAL and subsequently diluted to an OD_600_ of 0.1, which allowed the encapsulation of approximately one single cell per bead. Empty beads and beads carrying a high number of cells (initial cell culture OD ~ 1.4) were prepared as FC controls. A further control consisted of RW1 wild-type cells. All the material to be used (tubes, tips, pluronic acid) was preheated at 42°C and the procedure was carried out in a 37°C climate chamber. Fresh 2.5% agarose solution was prepared in deionized water and stored at 55°C, and transferred at 42°C only 20 min before starting the protocol. One ml of preheated 2.5% agarose solution was mixed with 30 μl of pluronic acid (Pluronic F-68 solution 10%, Sigma-Aldrich) by vortexing for 1 min. After that, 200 μl of RW1 cell or library suspension were added to the agarose solution and vortexed during one additional minute. A total of 500 μl of this agarose-cell mixture were transferred drop by drop into 15 ml of silicone oil (dimethylpolysiloxane, Sigma-Aldrich) preheated at 37°C while vortexing simultaneously (2 min). The tube was then immediately plunged into crushed ice and left for 10 min, after which it was centrifuged for 10 min at 2000 rpm. The oil was decanted, the beads were resuspended with 15 ml of PBS solution (phosphate buffered saline) and the residual oil was removed. The bead suspension was then passed through a sieve of 70 μm pore size and subsequently through a 40 μm-pore sieve, resulting in a <40 μm fraction (filtrate from second sieving) which was kept for microcolony growth screening.

**Figure 1:**
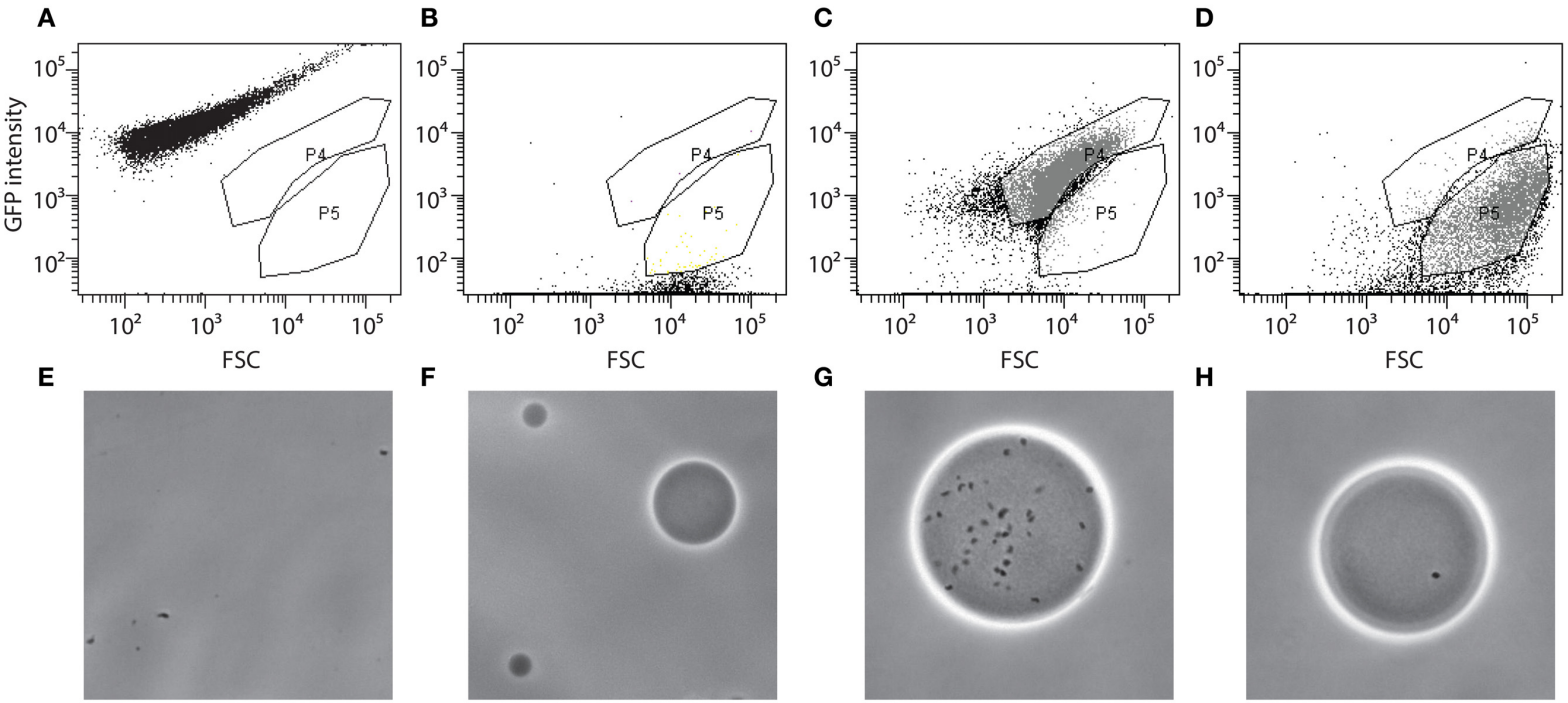
**Flow cytometer diagrams and corresponding microscope images of green fluorescence vs. forward scatter (FSC) of *S. wittichii* RW1 miniTn5 mutant cells embedded or not in agarose beads and stained with SYTO9**. MiniTn5 mutant library as free cells **(A,E)**, empty agarose beads **(B,F)**, agarose beads prepared with a highly concentrated cell culture OD_600_~1.4 **(C,G)**, or with a diluted cell culture OD_600_~0.07 **(D,H)**. P4, gate with beads with high cell density; P5, beads with low cell density.

Agarose beads containing RW1 mutant cells were analyzed by FC on a FACSAria (BD Biosciences) and using the BD FACSDiva software (version 6.1.3). An aliquot containing the cells-beads solution was stained by adding 1/1000 volume of SYTO9 solution (Invitrogen) and incubating in the dark for 15 min. The stained cell-bead mix was aspirated at approximately 50–100 μl/min and FSC, SSC and green fluorescence (FITC-channel) were recorded. Approximately 900,000 events were detected in the cell-bead initial mix. Gates were set using wild-type RW1 cell suspension (Figure 1A), a suspension of empty beads (Figure 1B), or beads prepared with RW1 cultures with an OD_600_ of 1.4 (Figure 1C) and 0.07 (Figure 1D). Gate P4 corresponds then to beads carrying a high cell number while P5 includes beads with a low cell density. The presence of free cells (Figure 1E), empty agarose beads (Figure 1F) and cells in beads from gates P4 and P5 (Figures 1G,H, respectively) was confirmed by sorting and subsequent epifluorescence/phase-contrast microscopy. After setting an accurate drop delay value (Accudrop protocol, FACSAria, BD Biosciences, Erembodegem, Belgium), P5 beads were sorted and recovered in a tube (Settings: Voltage FSC 25, SSC 383, FITC 429 / Threshold FSC 1000). The P5 subpopulation was then divided in three fractions. To one of those MM+Km was added (no carbon); to the second MM+Km+SAL (0.5 mM) and to the last one MM+Km+SAL+NaCl was added (to achieve a reduction in water potential of −1.5 MPa). The salicylate concentration (0.5 mM) was lower in this experiment to avoid microcolonies developing too large and escaping the beads. Bead suspensions were incubated at 30°C and 100 rpm for 3 days. A bead sample was analyzed for microcolony growth every day by staining, FC and epifluorescence microscopy. Gates were adjusted for FITC vs. SSC signals: Gate P1 corresponding to beads containing developed microcolonies (high fluorescence) and gate P2 corresponding to beads containing non-developed microcolonies (low fluorescence). Beads, which after 3 days of incubation entered in the P2-gate, were again sorted out individually and placed as microdroplets directly on MM+SAL+Km agar plates. Plates were incubated at 30°C until regular RW1 colonies were visible (~7 days). Transposon mutant colonies were then verified in liquid culture to determine growth rates and biomass yield in presence or absence of NaCl at −1.5 MPa.

**Figure 2:**
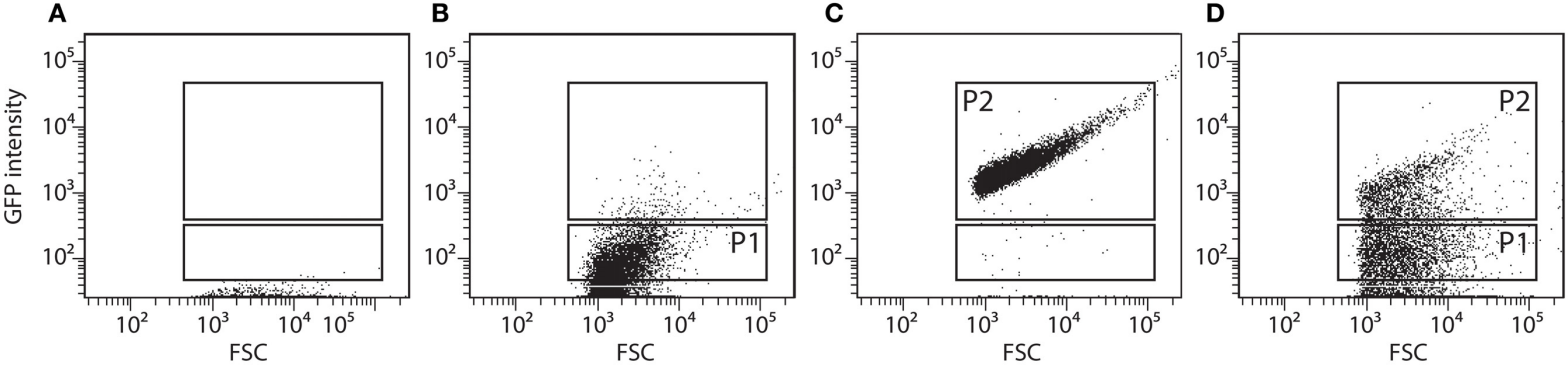
**Flow cytometer diagrams of green fluorescence vs. forward scatter (FSC) of *S. wittichii* RW1 wild-type cells (A), *E. coli* BW20767 (pRL27-*egfp*) (B), RW1 (miniTn5-*egfp*) with constitutively high eGFP production (C), and the uninduced RW1Tn5-*egfp* library (D)**. P1 was used as gate for low fluorescence whereas P2 was used to differentiate cells with high green fluorescence (see further **Figure 5** for explanation of the screening procedure).

The RW1 pRL27-*egfp* library was screened for cells producing a higher eGFP signal under growth conditions with decreased SP compared to the signal in control media. The assumption here was that an increased eGFP production under lower water potential would indicate that the insertion of the transposable element is within or close to a gene higher expressed under solute stress, and thus perhaps implicated in resisting this stress. A 1 ml aliquot of the library mix was taken out of the −80° storage, slowly thawed, diluted in 50 ml of fresh media (MM+SAL+Km, 5 mM) and incubated overnight at 30°C on a rotary shaker at 180 rpm. Single cell eGFP intensities in the library mutant cultures were determined by FC using the FITC-channel (FACSAria, BD Biosciences). Pure cultures of RW1 and *E. coli* BW20767 were employed to define the fluorescence level of cells not expressing eGFP (Figures 2A,B, P1 gate). An RW1 transposon mutant recovered from plate showing constitutive eGFP expression was selected to define the high fluorescence gate (Figure 2C, P2 gate).

To screen the RW1 mutant library, the P2 subpopulation had to be discarded, since it includes clones expressing eGFP constitutively. Thus, the P1 subpopulation was recovered by cell sorting (Settings: Voltage FSC 200, SSC 300, FITC 300/Threshold FSC 1000), transferred to an Erlenmeyer flask containing 20 ml of MM+SAL+Km and again incubated overnight with rotary shaking. This depleted mutant culture was then divided in two fractions. To one fraction (10 ml) 1 ml of a NaCl solution (174 g/L) was added to achieve a decrease in water potential of −1.5 MPa, whereas to the other 1 ml of sterile water (control) was added. The cultures were incubated on a rotary shaker and after 2 and 6 h of incubation, a 5 ml aliquot was taken from each flask to measure the fluorescence level of individual cells by FC. In this case, we focused on the cells having high eGFP fluorescence (P2 gate), assuming they might contain mutants with insertions near NaCl-inducible promoters. Cells in the P2-gate were sorted, transferred to new Erlenmeyer flasks containing 20 ml of MM+SAL+Km and grown until an OD_600_ of around 0.6. NaCl exposure was repeated once more and the cells falling into the P2 gate from the NaCl exposed cultures (both after 2 and 6 h) were again sorted. The recovered P2 cells were directly plated on MM+SAL+Km agar plates and incubated at 30°C until colonies developed. Individual colonies were picked up and transferred to 96-well microtiter plates containing 200 μl of MM+SAL+Km per well. In total 768 individual colonies in 8 microtiter plates were picked and kept as master plates. The master plates were used to inoculate two series of new microtiter plates, 8 plates containing control media (MM+SAL+Km) and 8 plates with NaCl-amended media (MM+SAL+Km+NaCl −1.5 MPa). The eGFP intensity and OD_600_ of NaCl-exposed plates were measured after 2, 4, 8, and 20 h using a FLUOstar Omega plate reader (BMG Labtech), and compared to those in the control plates (without NaCl addition). eGFP intensities were then normalized by the culture density. The growth rate of the different transposon mutant was determined.

### Identification of miniTn5 insertion sites

Total DNA of RW1 transposon mutants was extracted with the Xanthogenate method as described by Tillett and Neilan (2000). Briefly, overnight cell cultures were pelleted, resuspended in Xanthogenate lysis buffer (0.5 g Potassium Ethyl Xanthogenate, 10 ml of 4 M ammonium acetate, 5 ml of 1 M Tris-HCl pH 7.4, 2 ml of 0.45 M EDTA, 2.5 ml of 20% SDS solution, in a total volume of 50 ml H_2_O) and incubated at 65°C during 2 h. Cell debris was removed by centrifugation and the supernatant was transferred to a new tube into which one volume of phenol:chloroform:isoamyl alcohol (25:24:1) was added and mixed until emulsion formed. After centrifugation, the DNA in the aqueous phase was recovered, precipitated with isopropanol, then washed once with 70% ethanol, dried and finally resuspended in 200 μl of H_2_O. DNA was digested overnight in 20 μl with SacII (which does not cut inside the transposon), diluted to 100 μl, and treated with T4 DNA ligase to produce self-circularized fragments. This ligation mixture was transformed into *E. coli* DH5α λpir and plated on LB agar containing Km. Circularized fragments containing the transposed plasposon fragment replicate as plasmids because of the existing origin of replication (Larsen et al., 2002), and were purified from the *E. coli* transformants. Plasmid DNA was then used as template for BigDye® terminator sequencing according to the protocol of the supplier (Applied Biosystems), and using primers tnpRL17-1 for miniTn5 and tnpRL17-2 or GFPout for miniTn5-*egfp* (Table S3).

## Results

**Figure 3:**
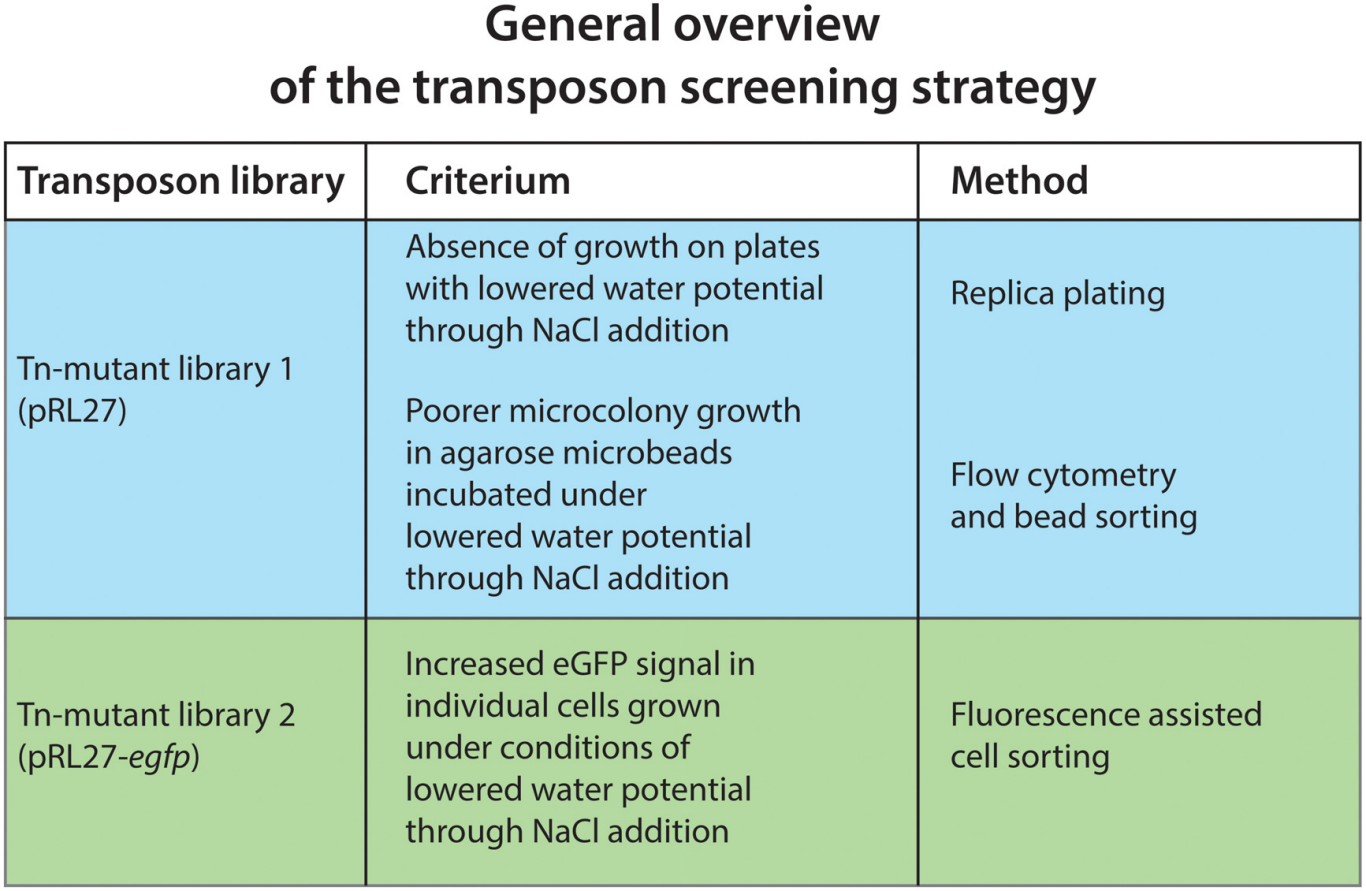
**General overview of the three Tn-mutant library screening procedures used in this study**.

We compared three screening procedures in order to recover and identify genes of RW1 potentially implicated in drought stress, which we mimicked by lowering the water potential of growth media by the addition of NaCl. The motivation for using different screening procedures was that drought stress has different facets, which we expected would be better covered by methods that each would emphasize a different screening aspect (i.e., absence of colony formation on plates, poorer growth in microbeads, gene expression). An overview of the three mutant screening procedures used in this study is depicted in Figure 3.

Two libraries of around 13,000 and 22,000 RW1 transconjugants were obtained in the conjugation procedure between *S. wittichii* RW1 and *E. coli* BW20767 (pRL27) as donor. To screen for mutants carrying a transposon insertion in essential genes for NaCl stress resistance, a total of some 2600 RW1 transposon mutant colonies were replica streaked on control medium plates and plates supplemented with NaCl to a calculated (additional) water potential decrease of −1.5 MPa.

Eleven colonies were found to be impaired in growth in the presence of NaCl and the insertion sites of the transposons were determined (Table 1). Two clones had an insertion in the gene designated Swit_2710, coding for a pseudouridine synthase C (clones 1-G3 and 3-G2), while other transposon insertions were located in genes Swit_2730, annotated as a hypothetical protein (clone 84), Swit_2731 coding for an aconitase-domain protein (clone 298), Swit_2958, coding for a BadM/Rrf2 family transcriptional regulator (clone 6-D11) and one with an insertion in the intergenic region between Swit_3114 and Swit_3115, which code for a hypothetical protein and ribosomal protein L36, respectively (clone 6-E3). The rest of the mutants had insertions in Swit_3468 (clone 355), an RNA polymerase β subunit, Swit_3770, coding for an AMP-dependent synthetase/ligase (clone 6-G5), Swit_4693, coding for a protein-disulfide isomerase-like protein (clone 7-D4), and finally, two clones with insertions in the intergenic region between Swit_5333 and Swit_5334 (clones 5 and 10-G5). These open reading frames code for a hypothetical protein and cell division FtsK/SpoIIIE, respectively.

**Table 1:** **Summary of growth rates (μ, h^−1^) in control media (Ctrl) and media with lowered water potential through NaCl-supplementation, of RW1 wild-type and selected recovered transposon mutant strains**.

**Clone**	**Insertion site**	**Transposon**	**Gene annotation**	**Maximum specific growth rate (h^−1^)**		**Ratio μ_Wild-type_/μ_mut_**		**Ratio μ_Ctrl_/μ_NaCl_**	**Ratio EGFP_Ctrl_/EGFP_NaCl_**	
				**Ctrl**	**NaCl**	**Ctrl**	**NaCl**		**4 h**	**8 h**
Wild-type		–		0.060±0.01	0.027±0.004					
Random Tn	Not determined	pRL27	Not determined	0.043±0.01	0.026±0.004					
3-G2	Swit_2710	pRL27	Pseudouridine synthase C, RluA family	0.024±0.0003	0.007±0.0008	2.2	2.7	3.3		
1-G3	Swit_2710	pRL27	Pseudouridine synthase C, RluA family	0.028±0.002	0.011±0.001	2.6	2.4	2.5		
84	Swit_2730	pRL27	Hypothetical protein	–	–	–	–	–		
298	Swit_2731	pRL27	Aconitase domain protein	–	–	–	–	–		
6-E3	Intergenic Swit_3114-Swit_3115	pRL27	Hypothetical protein Ribosomal protein L36, rpmJ	0.042±0.004	0.030±0.001	1.6	1	1.3		
6-D11	Swit_2958	pRL27	BadM/Rrf2 family transcriptional regulator	0.057±0.0009	0.026±0.0005	1	1	2.2		
355	Swit_3468	pRL27	RNA polymerase β subunit	0.044±0.002	0.029±0.001	1.1	0.8	1.5		
6-G5	Swit_3770	pRL27	AMP-dependent synthetase and ligase	0.033±0.001	0.017±0.0009	1.5	1.5	2		
7-D4	Swit_4693	pRL27	Protein-disulfide isomerase-like protein	0.053±0.005	0.027±0.001	1.3	1.1	1.9		
5	Intergenic Swit_5333-Swit_5334	pRL27	Hypothetical protein Cell division FtsK/SpoIIIE	0.035±0.0003	0.024±0.0003	1.4	1	1.4		
10-G5	Intergenic Swit_5333-Swit_5334	pRL27	Hypothetical protein Cell division FtsK/SpoIIIE	0.064±0.005	0.023±0.0009	1	1.35	2.7		
FACS26	Swit_5337	pRL27	GreA/GreB family elongation factor							
A1	Swit_3298	pRL27::egfp	Glyoxalase/bleomycin resistance/dioxygenase	0.047±0.001	0.037±0.004	1.3	0.7	1.2	1.1	1.3
B1	Swit_3298	pRL27::*egfp*	Glyoxalase/bleomycin resistance/dioxygenase	0.043±0.003	0.034±0.007	1.4	0.8	1.2	1.1	1.2
C3	Swit_3298	pRL27::*egfp*	Glyoxalase/bleomycin resistance/dioxygenase	0.040±0.009	0.047±0.001	1.5	0.5	1	1.3	1.5
F3	Swit_3298	pRL27::*egfp*	Glyoxalase/bleomycin resistance/dioxygenase	0.021±0.005	0.043±0.003	2.8	0.6	0.5	1.6	1.6
B12	Swit_3298	pRL27::*egfp*	Glyoxalase/bleomycin resistance/dioxygenase	0.049±0.006	0.037±0.004	1.2	0.7	1.3	1.2	1.2
H12	ND	pRL27::*egfp*		0.025±0.006	0.007±0.0008	2.3	3.8	3.6	1.2	1.3
G1	Swit_3298	pRL27::*egfp*	Glyoxalase/bleomycin resistance/dioxygenase	0.040±0.003	0.032±0.003	1.5	0.8	1.2	1.1	1.2
G8	Swit_4143	pRL27::*egfp*	Hydantoinase/oxoprolinase domain protein	0.042±0.004	0.039±0.004	1.4	0.6	1	1.1	1.1
H5	Swit_3298	pRL27::*egfp*	Glyoxalase/bleomycin resistance/dioxygenase	0.047±0.007	0.016±0.0007	1.3	1.7	3	1.2	1.3
D6	Swit_0265	pRL27::*egfp*	Glutamine amidotransferase	0.041±0.003	0.025±0.002	1.4	1	1.6	1.3	1.2
H10	Swit_4143	pRL27::*egfp*	Hydantoinase/oxoprolinase domain protein	0.040±0.004	0.027±0.0005	1.4	1	1.4	1.2	1.2
C7	Swit_3298	pRL27::*egfp*	Glyoxalase/bleomycin resistance/dioxygenase	0.042±0.0004	0.026±0.0003	1.4	1	1.6	1.2	1.1
A8	ND	pRL27::*egfp*		0.046±0.002	0.022±0.0014	1.3	1.2	2.1	1.1	1.2
F8	Swit 3298	pRL27::*egfp*	Glyoxalase/bleomycin resistance/dioxygenase	0.044±0.0004	0.025±0.0017	1.3	1	1.7	1.2	1.2
A9	Swit_3298	pRL27::*egfp*	Glyoxalase/bleomycin resistance/dioxygenase	0.046±0.0002	0.024±0.001	1.3	1.1	1.9	1.2	1.2
F1	Swit_3912	pRL27::*egfp*	Iron-sulfur cluster assembly accessory protein	0.046±9E-05	0.039±0.002	1.3	0.6	1.1	1.1	1.3

**Figure 4:**
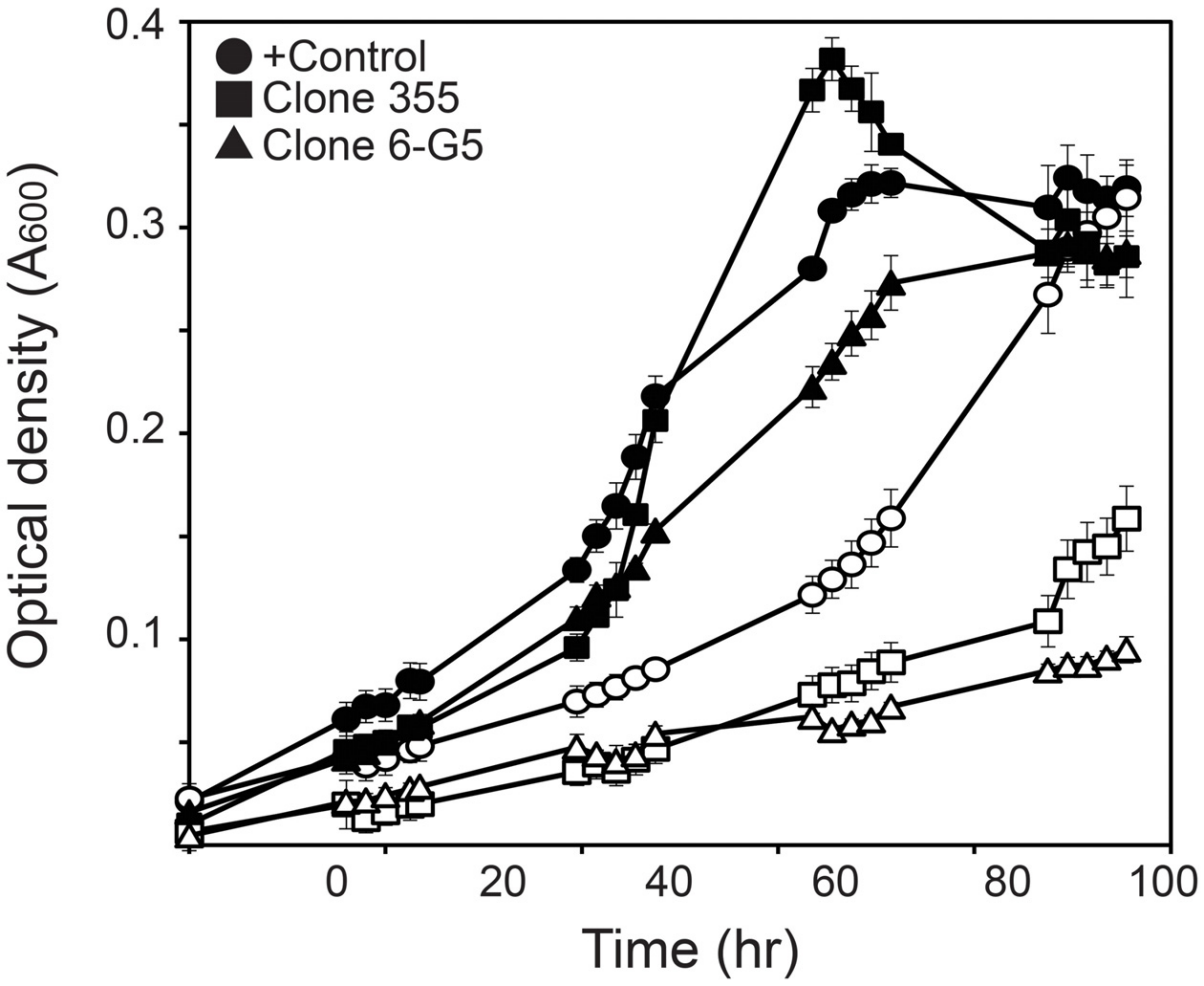
**Growth curves of transposon mutants growing on liquid control media (closed symbols) and media supplemented with NaCl (open symbols)**. Series correspond to a transposon mutant with no apparent growth impairment (Table 1, Random transposon mutant, •), a transposon mutant showing growth delay (Clone 355, ■) and a mutant characterized as salt sensitive (clone 6-G5, ▲).

While clones 84 and 298 were not able to regrow in liquid cultures, the growth rate of the rest of the mutants was determined by growth on MM+SAL+Km media and NaCl-supplemented media. The growth rate of the selected transposon mutants was indeed lower than the growth rate of the WT strain, both in control media and NaCl-supplemented media (Table 1). When comparing their growth curves, different growth patterns were distinguished. A group of four mutants (6-G5, 3-G2, 6-E3, and 1-G3) grew slower both in control and NaCl medium, reaching a low final OD_600_ compared to WT grown in both media (Figure 4). These were considered as salt-sensitive mutants. Five other mutants (10-G5, 7-D4, 6-D11, clone 5 and clone 355) showed a growth lag of 24 h in salt medium compared to WT and a random control Tn*5*-mutant. However, these mutants reached the same final OD_600_ on salt medium as the WT, but only after a longer time period. These mutants were characterized as slower growers in presence of NaCl (Figure 4).

**Figure 5:**
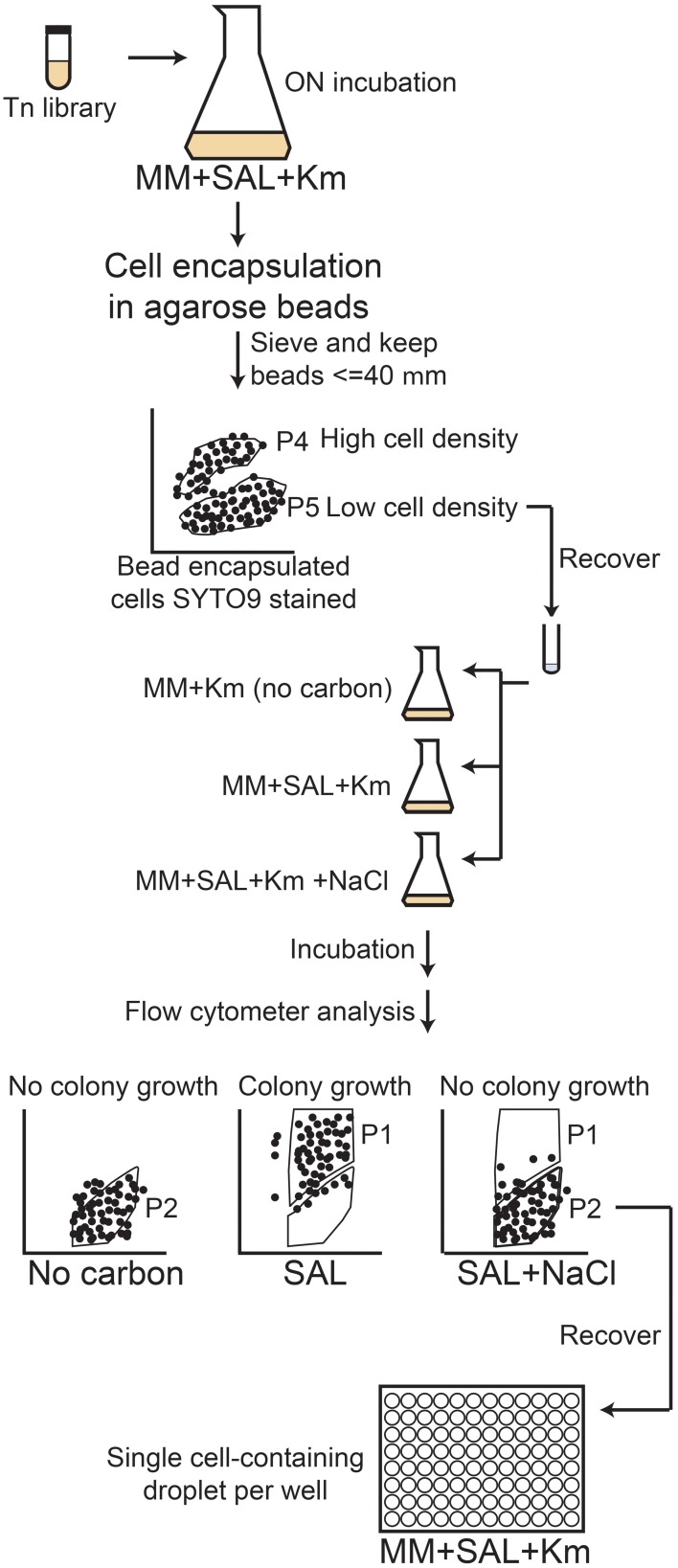
**Schematic diagram of the agarose encapsulation-flow cytometry screening procedure followed to detect RW1 transposon mutants with impaired water stress as a result of NaCl addition**. The agarose-encapsulated mutants were sorted to discriminate the beads carrying several cells (P4) inside the beads from the beads carrying one or few cells (P5). The P5 population was then exposed to no-carbon, SAL or SAL+NaCl conditions. After a 3-day incubation period, the beads were sorted to separate the high-fluorescence (high growth, P1) from the low-fluorescence (low-growth, P2) bead-embedded mutants. The low-growers were sorted out and their growth rates and biomass yields determined in presence or absence of NaCl.

**Figure 6:**
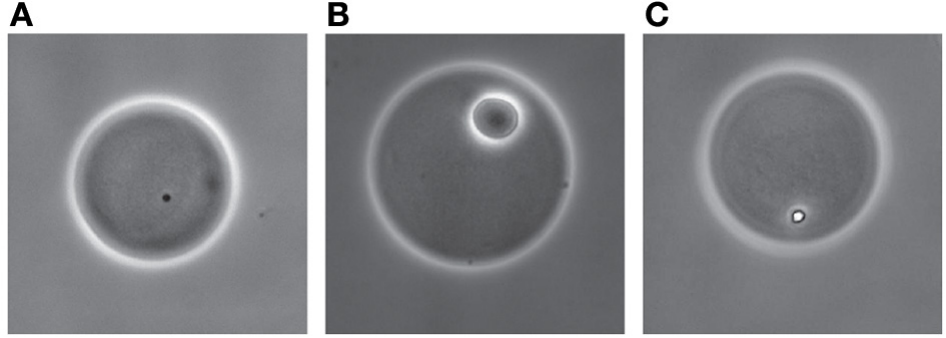
**RW1 microcolony growth inside agarose beads exposed to different media**. Bead with cells on MM with no carbon added **(A)**, in MM+SAL 0.5 mM **(B)**, or in MM+SAL 0.5 mM supplemented with NaCl **(C)**. Images show phase-contrast at 400× magnification.

This same mutant library was also screened by agarose bead encapsulation and by FC analysis and sorting, following a procedure illustrated schematically in Figure 5. After sorting nearly 900,000 cell-bead events, some 7200 beads of a size lower than 40 μm and mostly containing single cells were recovered. These beads were further exposed to conditions of no carbon, MM+SAL (0.5 mM) or MM+SAL (0.5 mM) supplemented with NaCl. This FC screening protocol allowed the selection of 400 clones that formed small microcolonies within beads in salt conditions (Figure 6C), comparable to those formed in media without any added carbon (Figure 6A). In contrast, much larger microcolonies formed in regular medium with SAL (Figure 6B). The clones were individually recovered on MM+SAL+Km (5 mM) agar plates. Thirty mutants of those developed into colonies on plate after sorting, and their growth rate was re-examined in MM+SAL+Km (5 mM) compared to MM+SAL+NaCl+Km (5 mM). Unfortunately, from the 30 recovered mutants, only one clone displayed repeatedly slower growth under salt conditions (Table 1, clone FACS26). The transposon insertion site was sequenced and this clone carries a mutation in the gene Swit_5337 (GreA/GreB family elongation factor), which is thought to interact with RNA polymerase for an efficient transcription. The mutant FACS26 showed a growth delay in salt liquid medium and the growth rate was lower in both control and salt media, compared to the WT strain in both growth conditions (Table 1). This mutant was thus characterized as a slower grower in salt conditions.

**Figure 7:**
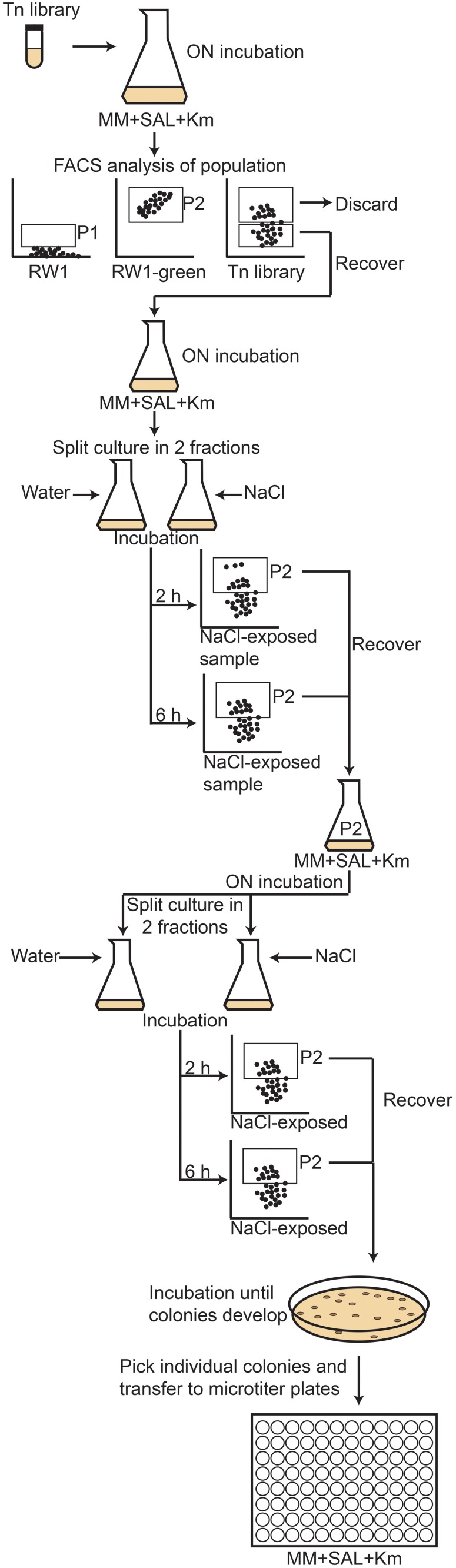
**Schematic diagram of the flow cytometry screening procedure followed to detect RW1 transposon mutants with an increased fluorescence as a result of NaCl addition**. The RW1 pRL27-*egfp* library was first sorted to separate the high fluorescence (P2) from low fluorescence clones (P1). The P1 subpopulation was then exposed to NaCl, incubated during 2 and 6 h and the fluorescence measured. The clones showing a high fluorescence after salt addition were recovered from gate P2 and plated on MM+SAL+Km. Once colonies developed on the plates, they were individually transferred to microtiter plates, where they were kept as master plates.

As an alternative to the traditional replica plating screening, which is a rather long and tedious process, and to the agarose beads screening, which gave us a very low recovery (only one clone consistently had a lower growth in salt media), a third screening method was developed by creating a new transposon mutant library using a modified pRL27 plasposon vector which carried a promoterless *egfp*. A new transposon library of around 22,000 mutants was produced by conjugation of RW1 and *E. coli* BW20767 (pRL27::miniTn5-*egfp*). In this case, the mutant library was screened for an increased eGFP signal in single cells when exposed to media with decreased SP (−1.5 MPa). The procedure followed is depicted in Figure 7.

**Figure 8:**
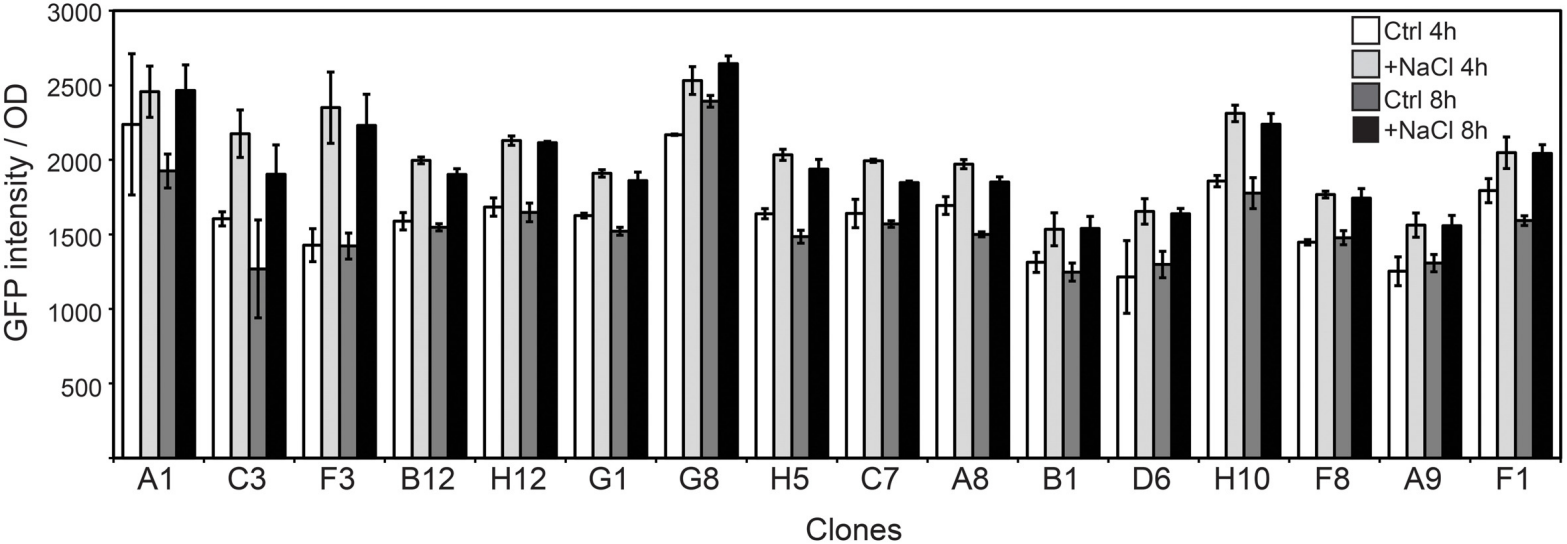
**Culture-density normalized eGFP values in selected RW1 mutants with consistent induction in medium with lowered water potential as a consequence of NaCl-amendment compared to control medium conditions without lower water potential (MM+SAL+Km)**. Measurements show values after 4 and 8 h of exposure.

The FC flow diagram of the RW1 mutant library showed that the library contains both cells with a low and a high fluorescence (Figure 2D). The cells falling into the P2 gate were discarded since we assumed they include mostly constitutively eGFP-producing clones. The cells in the P1 gate were sorted out and used to expose to growth conditions of lowered water potential by addition of NaCl. After two rounds of NaCl exposure and fluorescence-assisted sorting of potentially induced cells from P2, some 7000 cells were recovered from P2 and deposited on agar plates for culturing. 768 colonies were picked and rescreened in 96-well microtiter plate format to measure growth and fluorescence in the presence of NaCl compared to control conditions. A total of 45 mutant strains displayed a culture-density normalized eGFP signal 1.3–2 times higher when exposed to NaCl than in control media. After repeated verification, 16 of the 45 clones showed consistent higher normalized eGFP fluorescence when exposed to NaCl compared to the control (Figure 8). In some of those clones the signals developed only after 4 h and in others after 8 h of NaCl-exposure. On average, normalized eGFP signals in NaCl-induced cultures were between 1.3 and 1.6 times higher than in the control (Table 1).

Regarding their growth rates in salt media, one group of mutants (A1, B1, C3, F3, B12, G1, G8, and F1) showed a higher growth rate than the WT grown in the same NaCl-amended media. A second group of clones (H12, H5, A8, and A9) had a diminished growth rate in salt media when compared to the WT strain. A third set of mutants (D6, H10, C7, and F8) had a growth rate similar to the one observed in WT in salt exposure-conditions.

The mini-Tn5 insertion sites of 14 of the 16 clones were determined (Table 1). Clone H12 was not able to regrow and the sequence of clone A8 could never be recovered, despite numerous attempts. Ten out of the 14 recovered insertion sites were identical and had occurred within the gene Swit_3298, which codes for a protein from the family of glyoxalase/bleomycin resistance/dioxygenase. Two further clones were identical and had an insertion in the gene Swit_4143. This gene codes for a 5-oxoprolinase, which is involved in proline metabolism, catalyzing the interconversion of L-glutamate to 5-oxo-L-proline. One transposon insertion localized in Swit_0265, which is coding for glutamate synthase, involved in conversion of 2-oxoglutarate and L-glutamine into L-glutamate. The last clone identified carries an insertion in Swit_3912, which is annotated as an iron-sulfur cluster assembly protein.

## Discussion

Water stress has been considered as a major constraint in the survival of bacteria in the environment, which may limit the efficiency of strains introduced for bioremediation of toxic pollutants (Holden et al., 1997; Dechesne et al., 2010; Fida et al., 2013). Water stress resistance of bacterial strains may be a selection criterium for their use in bioaugmentation, but the factors enabling specific strains of interest for targeted pollutant degradation to cope with lowered water potential are insufficiently understood. Here we focused on an identification of genes possibly implicated in water-stress resistance by *S. wittichii* RW1, a representative of the large group of sphingomonads that degrade polycyclic aromatic hydrocarbons. Although stress by low water availability in e.g., a contaminated site is governed by a multitude of factors (Halverson and Firestone, 2000; Hallsworth et al., 2003; Or et al., 2007; Dechesne et al., 2008; Gülez et al., 2010), for simplicity of screening we focused here on water unavailability induced by the addition of NaCl. A water potential decrease of −1.5 MPa was selected as the condition for mutant library screening, since it diminished growth rates of RW1, but did not arrest growth completely (Johnson et al., 2011).

Resistance to genetic manipulation has so far hindered the creation of site-specific gene deletions and their complementation in RW1 (Coronado, unpublished), however, transposon mutagenesis by suicide plasmid delivery from *E. coli* donors is possible (Roggo et al., 2013). Hence, we used here three strategies to identify and obtain RW1 mutants with impaired water stress resistance through NaCl addition: in the first case we tested for reduced growth of mutants on agar plates with decreased water potential in comparison to control plates. As visual colony size comparison on plates (the basis for screening in replica plating) is prone to subjectivity, we designed a second method, which we hoped would have higher throughput and less subjectivity. In the second method, we screened for smaller-sized compared to average size microcolonies embedded in agarose beads grown in NaCl-supplemented media. Since this is quantifiable by FC signals (albeit not completely error-prone, because of bead-size variations), we expected more consistent mutant recovery. In the third strategy, we tested for increased expression of *egfp* in cells exposed to medium with lower water potential, with the idea that when the *egfp* transposon inserts in a gene that is higher expressed under lower water potential, it may be detected in the screen. In order to be able to do so, we used a different transposon mutant library, obtained with a mini-transposon to which a promoterless *egfp* was added. For these reasons, mutants recovered in the three methods are not necessarily congruent, because they come from different libraries and the screening focuses on different aspects of mutant growth behavior. However, it is surprising that all methods recovered different RW1 mutants, which were all impaired to some extent for growth under NaCl-lowered water potential media, but more overlap might have been found by increasing the library sizes and the numbers of screened mutants.

With the first mutant library screening, 11 clones were identified that could no longer grow on NaCl-amended agar plates (−1.5 MPa, Table 1). Two mutants carried the transposon in gene Swit_2710, but with slightly different insertion position of the transposon (~500 bp apart). Swit_2710 is predicted to code for a pseudouridine synthase C that belongs to the four-membered RluA family (RluA, RluC, RluD, and TruC). However, Swit_2710 only has direct homologs in genomes of sphingomonads (75–80% nucleotide identity in BlastN comparisons to the *nr* database). These enzymes are involved in the modification of uridine to pseudouridine (the C_5_-glycoside isomer of uridine) in RNA (Hamma and Ferré-D'amaré, 2006). Interestingly, an *E. coli* mutant with a truncated version of *rluD* could not form pseudouridine and showed poor growth (Gutgsell et al., 2001). The growth deficit was independent of pseudouridine depletion, which suggests that pseudouridine synthase possesses an additional function in growth regulation (Gutgsell et al., 2001). RluA has been found to be induced in conditions of high salinity in *Yersinia pestis* (Han et al., 2005), and Qiao et al. (2013) related the gene pseudouridine synthase to a stress response function. This supports the hypothesis that Swit_2710 pseudouridine synthase has a role in the resistance of RW1 to water stress conditions.

Two RW1 mutants were detected in the initial screen for NaCl-lowered water potential sensitivity, which, however could no longer grow in liquid MM+SAL+NaCl+Km medium (Table 1). One of those maps in Swit_2730 (coding for a hypothetical protein); the other in the upstream-located gene Swit_2731 (aconitase-domain containing protein). Swit_2730 and_2731 seem to be translationally coupled on the RW1 genome, but are the last two in a stretch of eight open reading frames all in the same transcription direction, making it hard to decide if they are co-transcribed or not. The two genes Swit_2730 and Swit_2731 are widely conserved in alphaproteobacteria (70–90% nucleotide identity by BlastN on the *nr* database), but not their immediate surrounding. Swit_2730 codes for a hypothetical protein. Johnson et al. (2011) found 10-fold lower expression of Swit_2730 in *S. wittichii* RW1 cultures grown on media amended with PEG to mimic matric stress compared to non-stressed medium. The downstream gene, Swit_2731 (aconitase-domain containing protein) could be involved in the TCA cycle catalyzing the reaction of isomerization of citrate into isocitrate via the intermediate *cis*-aconitate. In other organisms, the aconitase gene has been implicated in multiple functions other than TCA cycle. In *E. coli*, an aconitase gene is activated by the SoxRS oxidative stress regulatory system (Gruer and Guest, 1994; Cunningham et al., 1997) while a second aconitase is activated by the ferric uptake regulator (Gruer and Guest, 1994). In *Caulobacter crescentus*, an aconitase gene product was found to be part of a degradosome (Hardwick et al., 2011). In *Bacillus subtilis*, the CitB aconitase is both an enzyme and an RNA binding protein, and *citB* mutants are defective in sporulation, suggesting that the aconitase acts as an RNA binding regulatory protein (Serio et al., 2006). The interruption of Swit_2731, putatively involved in the TCA cycle, reduce the ability of RW1 to resist salt induced stress, suggesting that the metabolic activities in which it is involved, contribute to the resistance process.

One transposon insertion localized in gene Swit_2958. This gene encodes for a BadM/Rrf2 family transcriptional regulator. Interestingly, transposon insertions in Swit_2958 were also underrepresented in the mutant library cultured for 50 generations on salt medium (Roggo et al., 2013). Since this gene encodes a transcriptional regulator, it could perhaps modulate the expression of neighboring genes or of other genes important for water stress response. Directly upstream of Swit_2958 is a gene (Swit_2957) coding for an OsmC family protein. OsmC is induced by elevated osmolarity in *E. coli* and was speculated to have a peroxiredoxin activity working as scavenger for reactive oxygen species (Gutierrez and Devedjian, 1991; Shin et al., 2004). Although Swit_2957 itself was not identified as being differentially represented in the mutant libraries, another gene for an OsmC family protein (Swit_3232) was indeed underrepresented in the library grown for 50 generations on NaCl medium (Roggo et al., 2013).

One RW1 clone with reduced growth on medium with NaCl carried a transposon insertion in the intergenic region between Swit_3114 and Swit_3115. Swit_3115 encodes a ribosomal protein L36 that is part of the large subunit of the ribosome, which was shown as non-essential for protein synthesis or ribosome integrity in *E. coli* (Ikegami et al., 2005). Swit_3114 codes for a hypothetical protein and had already been identified as being 2.4 times up-regulated in a genome-wide transcription analysis of RW1 cells under a short-term perturbation with NaCl (−0.25 MPa) (Johnson et al., 2011). In addition, mutants in this intergenic region were underrepresented in the mutant libraries after 50 generations growth on salt medium (Roggo et al., 2013). All these results suggest that Swit_3114 plays a role when the cells have to deal with lower water stress. In contrast, both Swit_3114 and Swit_3115 were differentially regulated when *S. wittichii* RW1 was growing on dibenzofuran compared to cells growing on phenylalanine (Coronado et al., 2012). This suggests that they are also involved in other types of stress, such as exposition to a toxic compound. Fida et al. (2012) found an increased expression of the ribosomal protein L4 in response to chronic salt stress (the L4 protein is responsible for stabilizing mRNA and is related to stress response).

A further set of RW1 mutants was recovered with gene insertions that seem more remotely correlated to water stress resistance itself. One of these occurred in the gene Swit_3468, a gene with a size of 4368 bp, which codes for a RNA polymerase β subunit. The miniTn5 insertion is located 480 bp before the end of the gene. The RNA polymerase β could still be functional, but perhaps with a lower activity than the wild-type polymerase. The fact that the RNA polymerase β gene is interrupted, could explain the low growth rate observed in the transposon mutant with and without the exposure to NaCl. Secondly, an insertion occurred in Swit_3770, which is annotated as an AMP-dependent synthetase and ligase, but its true function is not known. This gene has similarity to the long-chain acyl CoA-synthetase from *Amycolatopsis mediterranei* (361 bp overlap), thus could be putatively involved in fatty acid biosynthesis.

One mutant contained a transposon in a gene coding for a protein-disulfide isomerase-like protein (Swit_4693). Protein-disulfide isomerases catalyze the structural change of disulfide bonds in proteins and play a role in proper protein folding. Therefore, Swit_4693 may have a role in maintaining folding of damaged proteins in cells exposed to salt stress. Finally, two clones were recovered with a transposon inserted in an intergenic region between the genes Swit_5333 and Swit_5334, but this insertion is unlikely to disrupt a promoter, because the genes are facing inwards. Swit_5333 and Swit_5334 encode, respectively, a hypothetical protein and the cell division protein FtsK/SpoIIE. This last one is a member of the division machinery that participates in the cell fission. A differential expression of an *ftsK* gene was demonstrated in *P. putida* KT2440 after exposure to 0.8 M urea, which was used to create a negative MP (Reva et al., 2006). However, since this gene has a general role in cell division, its function does not seem specific to water stress. Perhaps the phenotype of growth delay in liquid cultures supplemented with NaCl is caused by the transposon inserting in an uncharacterized gene within the 680-bp long intergenic region. This intergenic region contains a dozen of predicted ORFs, none of which has significant amino acid similarities with other sequences in the NCBI database.

The second procedure of library screening by agarose bead encapsulation, exposure to NaCl and sorting by FACS, contrary to our expectations only yielded one RW1 transposon mutant consistently growing slower in the presence of NaCl. We observed that of 400 selected beads only 30 grew to form microcolonies on agar plates. This suggests that either cells cannot escape very well from the agarose beads deposited on the agar surface, or were already damaged in the beads and could not regrow. When re-evaluating the growth of those 30 in control and NaCl-amended liquid cultures, only one clone showed consistent poor growth in salt-conditions. This clone (FACS26) had a transposon insertion in a gene for a GreA/GreB family elongation factor (Swit_5337), which interacts with RNA polymerase and stimulates the transcription elongation (Opalka et al., 2003). Despite the ease of producing and analyzing beads with cells, too many false-negative clones were picked up in the FACS procedure. False negatives may arise, for example, when clones grow poorly as a consequence of the sorting procedure, and not as a result of an SP decrease.

The third method relied on screening a RW1 mutant library that was produced by insertion of a modified transposon carrying a promoterless *egfp* by FC and cell sorting. This protocol allowed the recovery of 768 clones that produced higher eGFP intensity when exposed to NaCl. After two further rounds of NaCl exposure and eGFP measurement 14 mutants were recovered that consistently displayed higher normalized eGFP signals in the presence of NaCl-amended media. Interestingly, 10 clones carried the same insertion in gene Swit_3298, a protein from the broad family named glyoxalase/bleomycin resistance/dioxygenase, suggesting this mutant was abundant in the selected FC gates. The function of Swit_3298 is not known and the protein family comprises proteins with very broad activities. Swit_3298 has an amino acid similarity of 43% over the whole length to BphC (biphenyl-2,3-diol-1,2-dioxygenase) of several other organisms such as *Rhodococcus* sp. RHA1, *Rhodococcus globerulus* or *Mycobacterium tuberculosis*. On the other hand, the glyoxalase proteins are related to salt stress resistance factors in plants (Sairam and Tyagi, 2004; Lin et al., 2010).

Another transposon insertion with higher eGFP expression under NaCl-amended growth conditions occurred in Swit_3912, which belongs to the super family of iron-sulfur cluster assembly proteins. Proteins containing iron-sulfur clusters participate in a diversity of functions such as electron transport, substrate binding, regulation of gene expression and enzymatic activities (Johnson et al., 2005). It is not immediately clear what the function of gene Swit_3912 might be in resistance to salt stress.

Two further transposon insertions that resulted in higher eGFP expression under NaCl-amended growth conditions were located in Swit_4143 (putative 5-oxoprolinase) and Swit_0265 (putative glutamate synthase). Such enzymes are involved in the synthesis of proline and glutamate, which are known compatible solutes. As a consequence of hyperosmotic shock, the primary response in bacteria is to stimulate the uptake of potassium and synthesize glutamate (Sleator and Hill, 2002). The secondary response is the accumulation of neutral osmoprotectants (compatible solutes), which in contrast to the ionic osmolytes of the primary response, can be accumulated to high intracellular concentration to counteract the outflow of water, without adversely affecting cellular processes (Sleator and Hill, 2002). Compatible solutes also serve as stabilizers of proteins and cell components against the denaturing effects of high ionic strength (Kempf and Bremer, 1998; Sleator and Hill, 2002). Molecules such as glycine betaine, trehalose, glycerol, glucosylglycerol, proline, glutamate, ectoine, carnitine and choline, can be accumulated through synthesis or uptake from the environment following exposure to osmotic stress (Kempf and Bremer, 1998), with different microorganisms having a preference for one or more compatible solutes (Lucht and Bremer, 1994; Ogahara et al., 1995; Brill et al., 2011). In many microorganisms, proline biosynthesis proceeds from the precursor glutamate (Brill et al., 2011; Moses et al., 2012). In *B. subtilis* the accumulation of proline in osmotically stressed cells is followed by a decrease in glutamate level suggesting that *B. subtilis* prefers proline over glutamate as an osmolyte and begins to convert glutamate into proline as soon as it is exposed to osmotic stress (Brill et al., 2011). The results of our transposon mutant screening suggest that proline and glutamate are compatible solutes produced by RW1, important for the response of the cell to solute stress.

In summary, three mutant screening methods were applied to identify genes putatively involved in the water stress resistance induced by NaCl exposure by *S. wittichii* RW1. The three methods showed different efficiencies of relevant mutant recovery, but, surprisingly, the recovered mutants did not overlap between the three methods. The classical replica plating screening allowed recovering a higher proportion of mutants from the total of colonies screened. However this method requires picking several thousands of colonies in order to be exhaustive and long incubation times (up to 7 days for colonies to appear on agar plates), and visual screening of colony size differences is subjective. The screening involving FACS technology had a lower proportion of recovered mutants, however it permitted to screen a higher number of mutants in a shorter time period (up to 10^3^ per second), which makes it an interesting technique that needs further optimization. Despite being more quantitative, also in this case, the criterium of “poorer growth” in beads compared to normal growth, may not have been sufficiently strict to avoid many false-positives. Finally, in the third method we screened for eGFP expression of a transposon insertion, aiming that genes active under NaCl-induced lower water potential conditions would become detectable when hit by the mini-transposon. Since this is a different read-out, it could very well be that different mutants are recovered than in the two other screens that focus on reduced or absent growth.

As a consequence of the screening methods, we picked up several candidate genes that may not be regarded as typical salt or water stress genes. Several of the identified genes encoded hypothetical functions, while some of the genes with known function were involved in cell growth regulation (transcriptional regulator, elongation factor) and central metabolism (ribosomal protein, aconitase, pseudouridine synthase, iron-sulfur cluster assembly protein). Only two genes could be directly related to water stress, the genes Swit_4143 and Swit_0265, involved in proline and glutamate synthesis. Previous studies focused on strains relevant for bioremediation exposed to water stress found an increased degree of saturation of membrane fatty acids (Johnson et al., 2011; Fida et al., 2012), an up-regulation of the genes for biosynthesis or accumulation of compatible solutes such as trehalose (Johnson et al., 2011; Gülez et al., 2012), ectoine (Leblanc et al., 2008; Fida et al., 2012), glutamate (Moreno-Forero and Van Der Meer, 2014), of genes involved in oxidative stress (Leblanc et al., 2008), of exopolysaccharide-related genes (Gülez et al., 2012) and genes involved in DNA replication and repair (Leblanc et al., 2008; Johnson et al., 2011; Gülez et al., 2012) or genes involved in stress response (Leblanc et al., 2008; Gülez et al., 2012). Also, water stress can provoke the down-regulation of ribosomal proteins (Fida et al., 2012), of genes involved in motility (Johnson et al., 2011; Fida et al., 2012; Moreno-Forero and Van Der Meer, 2014), of transmembrane transporters (Reva et al., 2006) or outer membrane proteins (Fida et al., 2014). Our transposon mutant screening here indicated that in addition to specific water-stress response factors RW1 may need functions involved in general damage control when facing salt-induced water stress. Since no recombinant RW1 with improved survival in water stress conditions has been obtained yet, we suggest to pre-adapt the bacteria to low water activity conditions to improve their performance and survival in the field.

### Conflict of interest statement

The authors declare that the research was conducted in the absence of any commercial or financial relationships that could be construed as a potential conflict of interest.
