# Supplementary material for: Identification of genes potentially involved in solute stress response in Sphingomonas wittichii RW1 by transposon mutant recovery
Source: Front Microbiol. 2014 Nov 4;5:585. doi: 10.3389/fmicb.2014.00585 (PMC4219479; doi:10.3389/fmicb.2014.00585)
Supplement: Supplementary file 1 [file DataSheet1.DOCX]

Table S1. Bacterial strains used in this study.

| Strains |  | Description | Reference |
| --- | --- | --- | --- |
| *Sphingomonas wittichii* RW1 | | Dibenzofuran degrader | Wittich *et al*., 1992 |
| *E. coli* BW20767 | | For conjugative transfer of oriR6K oriT-RP4 plasmids. Has pir/ inserted into the chromosome. Donor of pRL27 plasposon. | Metcalf *et al.*, 1996; Larsen *et al*, 2002. |
| *E. coli* CC118λ*pir* | | For replication of pir-dependent plasmids | Herrero *et al*., 1990 |
| *E.coli*  S17-1λpir | | For replication and mobilization of plasmids with oriR6K. | de Lorenzo *et al*, 1990 |

Table S2. Plasmids employed in this study.

| Plasmids |  | Description | Reference |
| --- | --- | --- | --- |
| pRL27 |  | Codes for a hyperactive transposase and contains a miniTn5 -*ori* transposable element. Used for mutant library creation. | Larsen *et al*., 2002 |
| pRL27-gfp |  | For transposon mutant library creation, codes for a promoterless *egfp* gene in a miniTn5 transposable element | This study |

Table S3. Primers used in this study.

| Primer | Sequence (5'-3') | Description | Reference |
| --- | --- | --- | --- |
| tnpRL17-1 | aacaagcagggatgtaacg | Sequencing of miniTn5 insertion sites (pRL27) | Larsen *et al*., 2002 |
| tnpRL13-2 | cagcaacaccttcttcacga | Sequencing of miniTn5 insertion sites (pRL27-*egfp*) | Larsen *et al*., 2002 |
| GFPout | tcaacaagaattgggacaactccag | Annealing in *egfp* 70 nucleotides towards start | This study |
| npt-fw | atcgtggctggccacgacggg | Forward primer for the amplification of the Km resistance gene | This study |
| npt-rev | ctgatagcggtccgccacacc | Reverse primer for the amplification of the Km resistance gene | This study |
